# Ingredients, outcomes and mechanisms of change in complex interventions for children with movement limitations: a systematic evidence synthesis

**DOI:** 10.1186/1745-6215-16-S2-P5

**Published:** 2015-11-16

**Authors:** Niina Kolehmainen, Jennifer McAnuff, Annemarie Tissen-Budde

**Affiliations:** 1 Newcastle University, Newcastle, UK; 2 Pieter van Foreest, Hague, the Netherlands

## Background

Non-drug interventions for children with movement limitations lack evidence of effectiveness, and their evaluations are hindered by a lack of clarity about the interventions themselves.

## Aim

To synthesise literature about the proposed ingredients, outcomes and change mechanisms in two common non-drug interventions for children with movement impairments, namely occupational therapy (OT) and physiotherapy (PT).

## Method

Two electronic databases (PEDro, OTSeeker) were searched using ‘child’, ‘paediatric’ OR ‘adolescent’, limiting years to 2000-2011. Records were included if participants were 0-18-years-old with movement impairments; intervention involved OT/PT; AND primary outcome related to the child's activity/participation as specified by the WHO. Data were extracted on: study characteristics, intervention ingredients, intended outcome constructs, and hypothesised mechanisms. Two raters completed all screening and data extraction. Quantitative content analysis and descriptive statistics were used.

## Results

The included records (n=60, of n=918 screened) described n=52 studies, most focusing on children with cerebral palsy. Synthesis revealed n=33 intervention ingredients; 16/33 were common and corroborated across studies and in relation to wider literature (e.g. ‘practice’, ‘feedback’). n=43 intended primary outcomes, measured using n=41 instruments, were identified across the 52 studies. Hypothesised change mechanisms were mostly biomedical (48%); only 9/52 studies measured the mechanisms.

## Interpretation

The common, corroborated intervention techniques should be advanced to formal, robust evaluations. These evaluations will be more likely to result in cumulative learning if the field agrees to common core outcomes, and embeds systematic process evaluations (of hypothesised mechanisms) in the evaluations.

